# Circular RNA hsa_circ_0021001 in peripheral blood: a potential novel biomarker in the screening of intracranial aneurysm

**DOI:** 10.18632/oncotarget.22349

**Published:** 2017-11-10

**Authors:** Lingfang Teng, Yu Chen, Huihui Chen, Xijun He, Junyou Wang, Yujiang Peng, Hongyu Duan, Huiyong Li, Da Lin, Bo Shao

**Affiliations:** ^1^ Department of Neurosurgery, The First People’s Hospital of Wenling, Wenling 317500, Zhejiang Province, China

**Keywords:** circular RNAs (circRNAs), intracranial aneurysms (IA), disease-free survival (DFS), overall survival (OS), diagnosis

## Abstract

Circular RNAs (circRNAs) in the peripheral blood have been reported to be associated with cancer. However, there are few studies about circRNAs in intracranial aneurysms (IA). The purpose of the current study was to investigate the characteristic expression of circular RNA hsa_circ_0021001 in the peripheral blood of patients with intracranial aneurysms and its potential as a diagnostic biomarker for IA. In this study, a cohort of 223 cases of IA patients who were admitted in the department of neurosurgery in the First People’s Hospital of Wenling from January 2009 to July 2012 were collected as the experimental group, and 131 healthy volunteers over the same period served as the control group. Peripheral blood of each subject in both groups was collected on an empty stomach. The expression of hsa_circ_0021001 in peripheral blood was detected by real-time quantitative reverse transcription polymerase chain reaction (qRT-PCR) and the difference was analyzed by paired *t-test*. The effectiveness of hsa_circ_0021001 in the diagnosis of IA was assessed by ROC curve. Multivariate Cox proportional hazards regression analysis was used to analyze the prognosis. Hsa_circ_0021001 level in the peripheral blood of IA patients was relatively lower than that in the control group (P=0.002). The area under ROC (AUC) was 0.87, indicating that hsa_circ_0021001 was highly effective in the diagnosis of IA. In addition, hsa_circ_0021001 expression was correlated with aneurysm rupture, Hunt, Hess level, and timing of surgery (P= 0.041, 0.013, and 0.001, respectively). Patients with high expression of hsa_circ_0021001 had longer disease-free survival (DFS) and overall survival (OS) (P < 0.05). We found for the first time that hsa_circ_0021001 decreased significantly in the peripheral blood of IA patients, which suggested that hsa_circ_0021001 might be used as a potential novel marker for the diagnosis of IA.

## INTRODUCTION

Intracranial aneurysm (IA) is characterized by pathological dilatation intracranial artery as a pouch. Till now, IA rupture has been one of the most serious neurological diseases [1]. Early diagnosis of IA and prevention of rupture and bleeding are of important clinical significance. At present, diagnosis, prognosis, and evaluation of IA mainly depend on imaging, hemodynamics, and biological model construction [2]. Molecular biology studies have attracted more attention currently. The clinical manifestation of IA is abnormal dilatation of intracranial arteries, and thinning and weakening of the arterial wall by constantly impacting the vessel wall, thereby increasing the risk of aneurysm rupture [3]. IA occurs 2%-5% of the world population; and approximately 0.7%-1.9% of IA patients have ruptured, which cause life-threatening subarachnoid hemorrhage (SAH) [4]. IA is one of the most devastating neurological diseases due to its high mortality and poor prognosis. It is characterized by spontaneous intracerebral hemorrhage, cerebral vasospasm, and oculomotor nerve palsy [5]. Unfortunately, IA in most patients is asymptomatic until the tumor ruptures and pain appears and leads to subarachnoid hemorrhage [6]. Although surgery has improved, the prognosis of SAH hasn’t ameliorated in the past few decades, and the mortality rate is up to 40%, about half of the survivors are insufficiency [7, 8]. Hemodynamic disturbances, genetic abnormalities, infection, aging, and congenital factors are the common risk factors of the occurrence and progression of IA [9]. However, the pathogenesis of aneurysms remains unknown. In this context, it is urgent to identify biomolecules associated with the diagnosis and prognosis of IA.

Circular RNAs (circRNAs) are endogenous RNA with stable structure and highly tissue specific expression [10]. circRNAs are common in mammalian cells and regulate gene expression at the transcriptional or post transcriptional levels by interacting with microRNA or other molecules [11, 12]. circRNAs are different from linear RNAs because of remarkable non-standard splicing characteristics and no characteristic of free 3 ‘or’ 5 ’ends [13]. Recently, researchers have found that circRNAs are involved in the development of many diseases, such as atherosclerosis and nervous system diseases [14]. Present studies have shown that circRNAs have many functions, such as miRNA sponges, gene regulation, and close relation with cell function and diseases.

circRNAs end to end without free ends and can tolerate nuclease RNase R. Their structures are more stable than miRNA [15]. circRNAs widely spread in the blood, which makes them become potential noninvasive candidate biomarker resources [16]. However, little is known about their associations with cancers. In this study, we focused on a circRNA, hsa_circ_0021001 that locates at chr11:6499967-6501693 in circBase with its associated-gene symbol as ARFIP2 because hsa_circ_0021001 has been predicted to be associated with IA in circ2Traits (ID: hsa_circ_0021001 in database circ2Traits) 22. We first validated the expression of hsa_circ_0021001 in IA patients. Then we explored the role of hsa_circ_0021001 in the diagnosis and prognosis of IA. Our findings suggested that hsa_circ_0021001 may serve as a potential novel biomarker for IA.

## RESULTS

### Detection of hsa_circ_0021001 in IA patients

**Figure 1:**
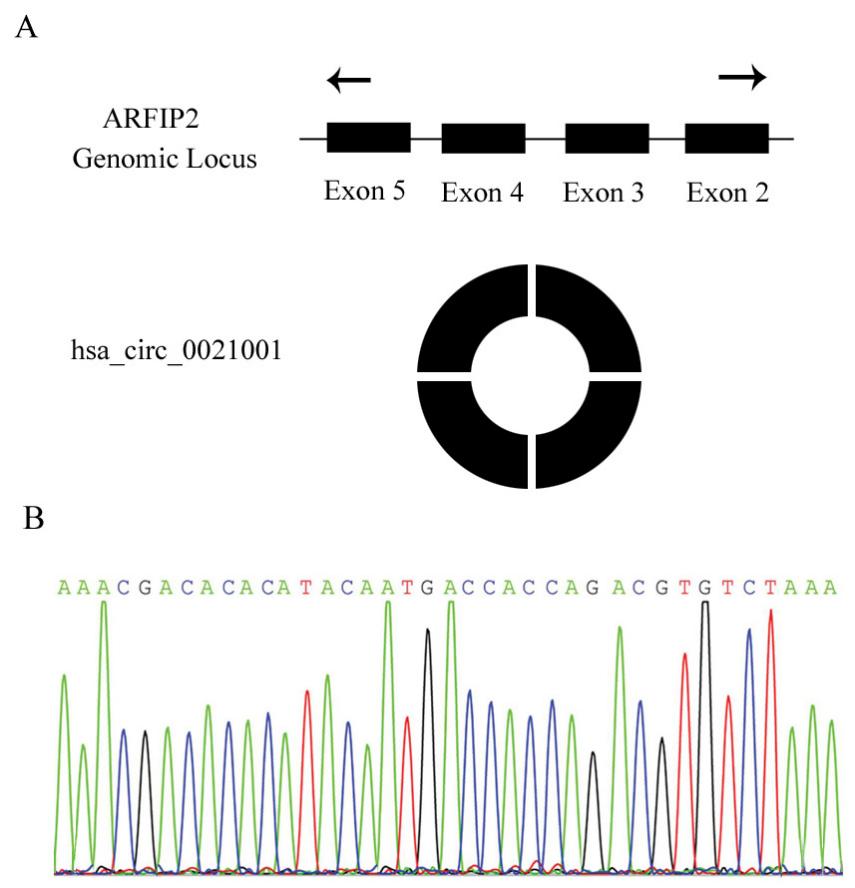
Identification of hsa_circ_0021001 in IA patients **(A)** hsa_circ_0021001 is produced at the ARFIP2 gene locus containing exon 2-5; **(B)** Sanger sequencing of hsa_circ_0021001 showed the back-splice junction.

Sanger sequencing confirmed the presence of hsa_circ_0021001 back-splicing site, demonstrating that hsa_circ_0021001 exists in a ring structure in nature (Figure 1).

### Down-expression of hsa_circ_0021001 in IA patients

**Figure 2:**
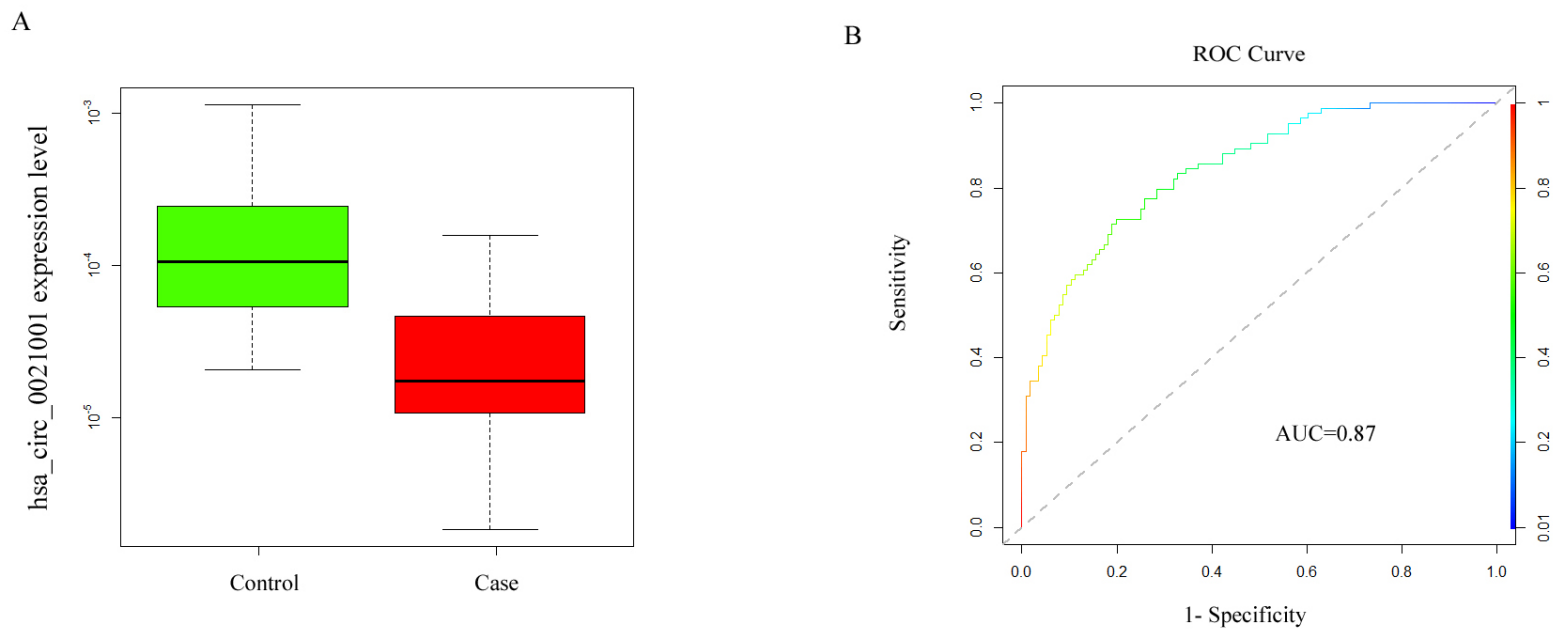
Hsa_circ_0021001 expression level in IA development **(A)** hsa_circ_0021001 expression in the experimental and control groups; **(B)** ROC curve for detection of the diagnostic efficiency of hsa_circ_0021001 in IA with an AUC of 0.87, a sensitivity of 0.81 and a specificity of 0.92. ROC curve: Receiver Operating Characteristic curve; AUC: area under the ROC curve.

In order to determine the difference in hsa_circ_0021001 expression between IA patients and healthy controls, total RNA of peripheral blood was isolated from 223 IA patients and 131 normal controls and analyzed by qRT-PCR. The expression of hsa_circ_0021001 in both groups was detected by qRT-PCR. Our results showed that hsa_circ_0021001 expression in IA patients was significantly lower than that in healthy controls (n = 131; P = 0.0014) (Figure 2A). The AUC of hsa_circ_0021001ROC was 0.87 (95% confidence interval (CI): 0.784-0.963). The specificity was 0.92 and sensitivity was 0.81, with diagnostic threshold of 20.74 (Figure 2B).

### The potential diagnostic value of hsa_circ_0021001 for IA

The results showed that the expression of hsa_circ_0021001 was significantly decreased in blood samples of IA, so we analyzed the relationship between hsa_circ_0021001 expression and the clinicopathological features of IA. As shown in Table 1, hsa_circ_0021001 expression was not significantly associated with age, gender, size of aneurysm, hypertension history, smoking, alcohol consumption, coronary heart disease, blood glucose, and aneurysm location (P>0.05). But, hsa_circ_0021001 expression was significantly associated with aneurysm rupture, Hunt Hess levels, and timing of surgery (P< 0.05).

**Table 1:** Association between hsa_circ_0021001 expression and clinicopathologic characteristics of intracranial aneurysm

Clinicopathologic parameter	Case number (n)	High hsa_circ_0021001 expression [n(%)]	Low hsa_circ_0021001 expression [n(%)]	χ2	P
Age (year)					
≥55	89	21 (23.6%)	68 (76.4%)	2.624	0.103
<55	134	18 (13.4%)	116 (86.6%)		
Gender					
Male	144	29 (20.1%)	115 (79.9%)	0.285	0.712
Female	79	9 (11.4%)	70 (88.6%)		
Aneurysm size (mm)					
≥10	80	10 (12.5%)	70 (87.5%)	0.370	0.617
<10	143	27 (18.9%)	116 (81.1%)		
History of hypertension					
Yes	97	22 (22.7%)	75 (77.3%)	2.758	0.226
No	126	17 (13.5%)	109 (86.5%)		
History of smoking and drinking				1.261	0.307
Yes	113	16 (14.2%)	97 (85.8%)		
No	110	23 (20.9%)	87 (79.1%)		
History of coronary heart disease				0.154	0.672
Yes	71	12 (16.9%)	59 (83.1%)		
No	152	27 (17.8%)	125 (82.2%)		
Blood glucose (mmol/L)				0.283	0.545
>5	80	13 (16.3%)	67 (83.7%)		
<5	143	27 (18.9%)	116 (81.1%)		
Aneurysm location				1.482	0.324
Anterior circulation aneurysm	196	31 (15.8%)	165 (84.2%)		
Posterior circulation aneurysm	27	5 (25.0%)	15 (75.0%)		
Rupture				9.018	0.002
Ruptured	162	19 (11.7%)	143 (88.3%)		
Unruptured	61	19 (31.1%)	42 (68.9%)		
Hunt-Hess grading				21.03	<0.001
Level I ∼ III	146	11 (7.5%)	135 (92.5%)		
Level IV ∼ V	77	26 (33.8%)	51 (66.2%)		
Surgical timing				13.16	0.005
Early-phase surgery	110	13 (11.8%)	97 (88.2%)		
Middle-phase surgery	68	7 (10.3%)	61 (89.7%)		
Late-phase surgery	45	13 (28.9%)	32 (71.1%)		

### Relationship between hsa_circ_0021001 and GOS in IA patients

According to our data, GOS was not significantly correlated with age, gender, tumor size, coronary heart disease, smoking, alcohol consumption, blood glucose and timing of surgery (P>0.05); while GOS was significantly associated with hypertension history, aneurysm location, rupture, Hunt Hess levels (P<0.05) (Table 2).

**Table 2:** Associations of GOS with hsa_circ_0021001 expression and clinicopathologic features of IA

Prognostic factors	n	Good prognosis [n(%)]	Poor prognosis [n(%)]	χ2	P
Age (year)				0.681	0.513
≥55	89	73 (82.0%)	16 (18.0%)		
<55	134	105 (78.4%)	29 (21.6%)		
Gender				3.115	0.231
Male	144	111 (77.1%)	33 (22.9%)		
Female	79	69 (87.3%)	10 (12.7%)		
Aneurysm size (mm)				0.782	0.503
≥10	80	67 (83.7%)	13 (16.3%)		
<10	143	113 (79.0%)	30 (21.0%)		
History of hypertension				12.304	0.002
Yes	97	90 (92.8%)	7 (7.2%)		
No	126	90 (71.4%)	36 (28.6%)		
History of smoking and drinking				5.381	0.072
Yes	113	98 (86.7%)	15 (13.3%)		
No	110	83 (75.5%)	27 (24.5%)		
History of coronary heart disease				0.683	0.504
Yes	71	58 (81.7%)	13 (18.3%)		
No	152	125 (82.2%)	27 (17.8%)		
Blood glucose (mmol/L)				3.320	0.265
>5	80	59 (73.8%)	21 (26.2%)		
<5	143	113 (79.0%)	30 (21.0%)		
Aneurysm location				14.644	<0.001
Anterior circulation aneurysm	196	173 (88.3%)	23 (11.7%)		
Posterior circulation aneurysm	27	13 (48.1%)	14 (51.9%)		
Rupture				15.87	<0.001
Ruptured	162	138 (85.2%)	24 (14.8%)		
Unruptured	61	32 (52.5%)	29 (47.5%)		
Hunt-Hess grading				11.06	0.003
Level I ∼ III	146	127 (87.0%)	19 (13.0%)		
Level IV ∼ V	77	53 (68.8%)	24 (31.2%)		
Surgical timing				0.404	0.970
Early-phase surgery	110	89 (80.9%)	21 (19.1%)		
Middle-phase surgery	68	56 (82.4%)	12 (17.6%)		
Late-phase surgery	45	37 (82.2%)	8 (17.8%)		
hsa_circ_0021001				6.866	0.007
High expression	39	26 (66.7%)	13 (33.3%)		
Low expression	184	157 (85.3%)	27 (14.7%)		

GOS, Glasgow Prognosis Score; IA, intracranial aneurysm.

### Results of follow up and risk factors

**Figure 3:**
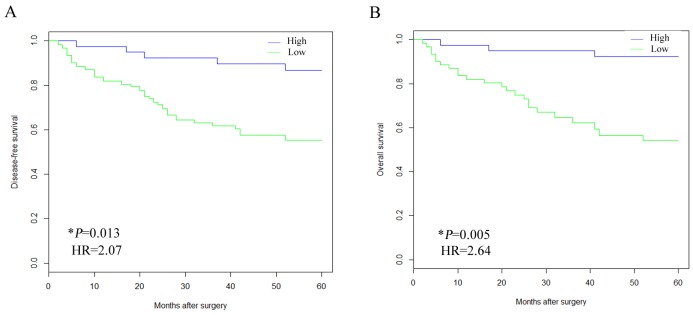
Kaplan-Meier analysis of survival rate of IA patients according to the expression status of hsa_circ_0021001 **(A)** association between hsa_circ_0021001 expression and DFS, P<0.05 in the Log-rank test; **(B)** Association between hsa_circ_0021001 expression and OS, P<0.05 in the Log-rank test. DFS: disease-free survival; OS: overall survival.

As shown by the survival curve, 8 cases were lost follow-up during the five years. DFS and OS were significantly longer in IA patients with higher expression of hsa_circ_0021001 than those with low expression (DFS: 33.1 vs 26.9, P = 0.013; OS: 32.2 vs 25.1, P = 0.005) (Figure 3). The Cox regression model showed that hsa_circ_0021001 expression, aneurysm location, rupture, and Hunt-Hess levels were the prognostic risk factors for IA (Table 3).

**Table 3:** Risk factors to IA prognosis by multivariate Cox proportional hazards regression analysis

	B	SE	Wald	df	Sig.	Exp (B)	95.0% CI for Exp (B)	
							Lower	Upper
Hsa_circ_0021001 expression	2.307	1.036	4.586	1	0.03	9.371	1.147	67.051
Hypertension	0.335	0.286	0.642	1	0.503	1.365	0.692	2.33
Aneurysm location	1.306	0.304	15.334	1	<0.001	3.810	1.774	7.024
Rupture	1.892	0.815	8.067	1	0.007	7.226	1.641	28.631
Hunt-Hess grading	1.130	0.310	11.283	1	<0.001	2.714	1.503	5.408

IA, intracranial aneurysm; B, regression coefficient; SE, standard error of regression coefficient; Wald, test statistics; df, degree of freedom. Sig., significance; Exp (B). index of the regression coefficients; CI, confidence interval.

## DISCUSSIONS

Our results showed that hsa_circ_0021001 expression was significantly lower in IA patients than that in healthy controls. The expression of hsa_circ_0021001 was significantly associated with aneurysm rupture, Hunt Hess levels, and timing of surgery (P<0.05). Moreover, we found that IA patients with high expression of hsa_circ_0021001 had longer DFS and OS than patients with low hsa_circ_0021001 expression. Our results further suggested that downregulation of hsa_circ_0021001 was associated with shorter survival of IA patients. ROC analysis proved that hsa_circ_0021001 can be used as a biomarker for IA with high accuracy, sensitivity, and specificity.

Circular RNAs were found to act as competitive endogenous RNAs binding proteins (protein sponges) [17, 18]. circRNAs regulate gene expression by a variety of mechanisms: some circRNAs can act as microRNA (miRNA) sponges, playing a competitive role in binding miRNA by post transcriptional regulation [19]; some circRNAs can also modulate transcription by interacting with nuclear small RNA (snRNA) or RNA polymerase II [20]; some circRNAs can modulate RNA splicing by binding to transcription factors [21]. circRNAs play an important role in various diseases, such as cancer, atherosclerosis, arthritis, pulmonary fibrosis, myotonic dystrophy, and Alzheimer’s disease [22–24]. For example, hsa_circ_002059 can be used as a new biomarker for gastric cancer [25]. circRNAs are used as clinical diagnosis tools for esophageal carcinoma [26]. Hsa_circ_0005075 can be a potential biomarker for hepatocellular carcinoma [27]. In this study, we focused on hsa_circ_0021001 and found its potential RNA binding protein (RBP) binding site (starBase, V2.0). These results indicated that hsa_circ_0021001 participates in and promotes the development of IA as protein sponge or transcription regulator. Two circRNAs have been proved to be miRNA sponges in mammals [25, 28]. A specific circRNA (cirs-7, also known as CDR1as), has more than 70 binding sites of miR-7. circRNA has been reported to impair miR-7 regulation *in vivo*. The second circRNA is a testis specific transcript which can determine the expression of male determining gene SRY. It contains 16 binding sites of miR-138. Many miRNA binding sites on circRNAs are predicted to have function. These results imply that hsa_circ_0021001 may play an important role in tumorigenesis and metastasis of IA by interaction with miRNA.

In conclusion, we first identified a significant decrease in the expression of hsa_circ_0021001 in IA patients, which may serve as a potential novel biomarker for IA. Moreover, our findings suggest that hsa_circ_0021001 may be involved in the genesis and metastasis of IA.

## MATERIALS AND METHODS

### Study population

A cohort of 223 cases of IA patients who were admitted in the department of neurosurgery in the First People’s Hospital of Wenling from January 2009 to July 2012 were collected as the experimental group, and 131 healthy volunteers over the same period served as the control group. After admission, all patients underwent computed tomography (CT) examinations and diagnosed by three-dimensional computed tomography angiography (3D.CTA) or digital subtraction angiography (DSA) before surgery. Exclusion criteria: patients suffering from other serious cardiovascular or cerebrovascular diseases; patients with other serious organs diseases; patients with incomplete clinical data. 223 cases of IA patients included 141 males and 82 females. The age range was 20-77 years, and the average age was 52 years. The imaging data of IA patients in this study showed that the diameter of the aneurysm <5 mm in 38 cases, between 5 mm-10 mm in124 cases, more than 10 mm in 61 cases, anterior circulation aneurysms in 204 cases, posterior circulation aneurysms in 19 cases; ruptured aneurysm in 130 cases, unruptured aneurysm in 80 cases. There was no significant difference in age, gender, blood pressure, blood sugar, blood lipid, and smoking between the experimental group and the control group (Table 1). Blood pressure examination included hypertension history, treatment, systolic and diastolic pressure, mean pressure at admission. Blood sugar examination included diabetes history, blood sugar abnormality history, treatment, and fasting blood sugar at admission. Blood lipids examination included hyperlipidemia history, treatment, triglycerid, cholesterol, high-density lipoprotein, and low-density lipoprotein at admission. Smoking history included a calculation of the average annual smoking amount based on smoking years and the number of smoked cigarettes per day. All participants signed written informed consent.

### Blood sample collection

Blood samples were collected at fasting in the early morning by a vacuum collecting vessel, then stored at 4°C. They were processed in 6 h. Samples were centrifuged at 1600 g/min, 12 min, and then blood cell components were removed. The upper plasma/serum were transferred into a new sterile RNase-free centrifuge tube, centrifuged at 14000 g/min, 16 min. Large granular material such as cell debris was removed, and the upper plasma/serum was transferred into a new 1.5 ml RNase-free centrifuge tube and stored immediately at -80°C.

### Total RNA extraction

RNA was extracted from the whole blood plasma according to the manufacture’s instruction (QIAGEN, Valencia, CA, USA). Then the optical density (OD) 260/280 was determined by ultraviolet spectrophotometry. After calculating the concentration, RNA was frozen in -80°C refrigerator.

### Detection of hsa_circ_0021001 expression by real-time quantitative PCR

qRT-PCR experiment was performed using GoTaq qPCR Master Mix kit and Mx3005P real-time PCR operating system. According to the procedures of the kit, 25 μL reaction systems consisted of 5 μL cDNA products, 5.5 μL DEPC water, 1 μL upstream primers, 1 μL downstream primers, and 12.5 μL qPCR mixtures. Hsa_circ_0021001 primers and GAPDH primers were synthesized by Shanghai Sangon biotechnology Inc. The primer sequences of hsa_circ_0021001 were as follows: 5′-GAAACTCGAGCCGCGCTGCGATATGTG-3′ (upstream); 5′-CACAGCCAGCAAAGTTACTCGCTTTAAA-3′ (downstream). GAPDH was used as an internal standard. The primer sequences of GAPDH were as follows: 5′-CCCGATAACACAAGTGCAGC-3′ (upstream); 5′-CCCGATAACACAAGTGCAGC -3′ (downstream). The relative expression of hsa_circ_0021001 was calculated using 2^−ΔΔCt^. All data were expressed as mean ± SD of three independent experiments. The experiments were performed by blind method.

### Sanger sequencing

The target fragment was inserted into a T vector and the length was determined by Sanger. The following primers were designed by Shanghai Sangon biotechnology Inc to test the back-splicing site of hsa_circ_0021001: 5′- CAATGCTGAAAACTGCTGAGAGAAG-3′ (upstream); 5′-CCTGCATTCTCTTTTCTGTTGTATCTTTAA-3′ (downstream).

### Prognosis and follow-up

The follow-up was conducted by telephone and clinical for 3 years. The prognosis after surgery was determined by Glasgow prognostic score (GOS). The follow-up was from the hospital discharge time after the patient was treated to January 2015. For survival patients at the end of the follow-up visit, the follow-up data were the last contact state. For patients lost follow-up, the follow-up data were the last census state. The survival time was expressed by survival months. Overall survival (OS) was the duration from aneurysm neck clipping to the death of the patient, and the disease-free survival (DFS) was the period at which the operation started without recurrence or death due to IA.

### Statistical analysis

All data were analyzed by R software (V, 2.15.0, Http://www.r-project.org/). The difference in hsa_circ_0021001 expression between IA patients and normal healthy controls were analyzed by paired *t-test*. The relationship between hsa_circ_0021001 and clinicopathological factors was evaluated by one-way ANOVA. ROC curve was drawn to access the diagnosis value. Cutoff value of hsa_circ_0021001 was analyzed by SigmaPlot 12.3. The survival curve was drawn by Kaplan–Meier method and analyzed by Long-rank test. Multivariate Cox proportional hazards regression analysis was used to analyze the prognosis. P<0.05 was considered statistically significant. The experiment repeatability was determined by Pearson correlation test.

## Abbreviations

circRNAs: circular RNAs
IA: intracranial aneurysms
DFS: disease-free survival
OS: overall survival
AUC: area under ROC
SAH: subarachnoid hemorrhage
